# How to demonstrate clinical implication of 7‐day continuous electrocardiographic patch monitoring

**DOI:** 10.1002/joa3.12923

**Published:** 2023-08-29

**Authors:** Naoya Kataoka, Teruhiko Imamura

**Affiliations:** ^1^ Second Department of Internal Medicine University of Toyama Toyama Japan

## CONFLICT OF INTEREST STATEMENT

Authors declare no conflict of interests for this article.

*To editor*:

Detection of lethal arrhythmia is of great importance in the diagnosis of patients with palpitations. Various noninvasive devices have been developed to monitor and detect critical arrhythmias. Kim and colleagues demonstrated that a 7‐day patch‐type continuous electrocardiogram (ECG) monitor (MEMO patch®) had acceptable feasibility and efficacy in detecting arrhythmias when compared with conventional 24‐h Holter ECG monitoring.^1^ Several concerns have been raised.

Various long‐term ECG monitoring devices have demonstrated their efficacy so far.^2^ The novelty of this study is unclear. In their study, the rates of clinically important arrhythmias were not significantly different between the two devices,^1^ and further larger‐scale studies are warranted to validate the clinical utility of MEMO patch.

One of the issues of these monitoring devices is their water resistance. If the MEMO patch is not water resistant, it cannot be used during bathing, when the autonomic nervous system fluctuates dramatically and the risk of critical arrhythmias increases.

The ability to detect arrhythmias depends on the duration of monitoring.^2^ It may be unfair to compare 7‐day monitoring with MEMO path and 1‐day monitoring with conventional Holter devices.^1^ Instead, it would be ideal to monitor patients' arrhythmias with two devices simultaneously during the same observation period.

It is generally challenging to automatically distinguish atrial fibrillation from sinus tachycardia by detecting R‐R irregularity. Was the presence of atrial fibrillation detected automatically or manually by using MEMO patch?
